# Supplementary material for: Improved detection of microbiome-disease associations via population structure-aware generalized linear mixed effects models (microSLAM)
Source: PLoS Comput Biol. 2025 May 27;21(5):e1012277. doi: 10.1371/journal.pcbi.1012277 (PMC12136445; doi:10.1371/journal.pcbi.1012277)
Supplement: S1 Text — (DOCX) [file pcbi.1012277.s001.docx]

**S1 Text – Additional Methodological Details**

### Genetic Relatedness Matrix

We used the pairwise Manhattan similarity (1 – Manhattan distance) based on the gene presence/absence matrix to create the Genetic Relatedness Matrix (GRM) for a set of samples. This is computed for all pairs of samples where a given species is present to create that species’ GRM $\psi$.

Users can substitute any kind of pairwise similarity matrix. For example, one could instead use a single-nucleotide variant (SNV) presence/absence or frequency matrix instead of a gene presence/absence matrix to compute the GRM. For SNV distance, we recommend using the Manhattan distance. The resulting GRM based on the set of bi-allelic polymorphisms genotyped by MIDAS v3 or another tool would approximate the average nucleotide identity (ANI) between samples. We explored this approach and found that polymorphism based GRMs were generally very different from gene presence/absence based GRMs for the same species. In simulations, this led to higher false positive rates for the microSLAM β test (similar to glm), presumably because there was trait-associated population structure in the gene presence/absence matrix for which this approach did not fully adjust. Further investigation into the selection of SNVs or distance metric could potentially make this strategy more effective.

### Microbiome generalized linear mixed model for binary traits: additional details about microSLAM’s modeling approach

In a case-control study with sample size N, we denote the status of the $i$th individual with $y_{i}=1$ or $0$, depending on whether it is a case or a control. Let the $1\times(1+p)$ vector $X_{i}$represent $p$ covariates, plus an intercept term, and let $G_{i}$ represent the presence or absence of a gene; this can also be replaced with the copy number of a gene, as estimated by MIDAS v3 or other tools. The logistic mixed model can be written as:

$logit(\mu_{i})=X_{i}\alpha+G_{i}\beta+b_{i}+\varepsilon_{i}$

where $\mu_{i}=P(y_{i}=1|X_{i},G_{i},b_{i})$ is the probability that the $i'$th individual is a case given the covariates, gene presence/absence vector, and the random effect $b_{i}$ that is estimated by microSLAM. The random effect $b_{i}$ is modeled $N(0,\tau\psi)$ where $\psi$ the N×N GRM described above, and $\tau$ is the estimated additive genetic variance. The $Var(y_{i}| b )=\phi var(\mu_{i})$, in the case of a binary trait the random parameter Φ=1. The parameter 𝛼 is a $1\times(1+p)$ coefficient vector of fixed effects and $\beta$ is a coefficient representing the log odds ratio for the association between the gene’s presence and the trait. For a quantitative trait, $y_{i}$ is a real number and the model is a linear mixed model rather than logistic, so that $\beta$ represents the expected change in the trait for the gene being present versus absent. Everything else is the same.

### Estimating the coefficients and variance component: fitting microSLAM for each species

We employ the same restricted log-likelihood and average information matrix for estimating the coefficients and variance components as were employed in GMMAT [1] and SAIGE [2]. For more details on deriving these estimation procedures, refer to those studies and to Clayton and Breslow [3]. We also follow SAIGE’s multi-step process to estimate the random effects and then use these in the logistic (or linear) model presented in the previous section. This helps us in two ways: 1) it reduces computational time significantly as random effects only have to be estimated one time for each species (not once for every gene in every species), and 2) avoiding refitting the random effect for every gene provides a more robust estimate.

Unlike SAIGE, we do not use PCG, randomized trace estimator, or a low-rank GRM. These are designed to reduce computation and memory costs within the context of human genomes with millions of genetic variants, but these are not major problems for us given the size of the datasets in this study. Also, our GRMs are naturally full-rank. These computational shortcuts could be implemented if needed.

### Score testing for the GRM: 𝛕 test modeling

We detail microSLAM’s 𝜏 test, a statistical procedure to inform the user whether the species' GRM is significantly related to the trait. This would indicate that a subset of related strains can predict the trait. We consider random effects $b_{i}\sim N(0,\tau\psi)$, as described above, then compare the models:

$$H_{0}: Y=X\alpha+\epsilon$$

$$H_{a}: Y=X\alpha+b+\epsilon$$

After the models have been fit (estimation converges), we have $\hat{\alpha}$; $\hat{b}$; $\hat{\phi}$; and $\hat{\tau}$. We also compute a working vector

$$\hat{Y}=X \hat{\alpha} + \hat{b}+ \hat{\epsilon}, b\sim N(0,\hat{\tau} \psi); \hat{\epsilon} \sim N(0,W^{-1})$$

The test statistic for the 𝜏 test can be written as:

$$T=\sum_{i=1}^{N} \hat{{b^{2}}_{i}}/N$$

This is the sample variance of the estimated random effects $b_{i}$. This statistic involves the sum of the squared random effect estimates. The null hypothesis is that $\tau$=0 (i.e., the random effects do not help to explain variation in the trait). To compute a p-value for $T$ without making assumptions about its distribution, we use a permutation test.

### Score testing for gene presence/absence: 𝜷 test modeling

After we have fit the model described above for the 𝜏 test, we have estimates of the fixed effect coefficients $\hat{\alpha}$, the random effects $\hat{b}$, and the variance component parameters, $\hat{\phi}$; $\hat{\tau}$. Using these, we construct a score test for each gene with the null hypothesis $H_{0} :\beta=0$. Suppose $G$ is a $N \times1$ genotype vector (where $N$ is the number of samples). $\hat{\mu}$ are the probabilities of the samples having the trait (e.g., being cases) given the covariates $X$ and the random effects $\hat{b}$: $P(Y=1|X,\hat{b})$. As in SAIGE, we let $\hat{W}$be a diagonal vector with elements $\hat{\mu} (1-\hat{\mu} )$and $\tilde{G}=G-X(X^{t}\hat{W}X)^{-1}X^{t}\hat{W}G$ is the covariate-adjusted genotype vector. With $\hat{\Sigma}=\hat{W^{-1}}+\hat{\tau}\psi$ and $P=\hat{\Sigma^{-1}}-\hat{\Sigma^{-1}}X(X^{t}\hat{\Sigma^{-1}}X)^{-1}X^{t}\hat{\Sigma^{-1}}$ and a working vector $\hat{Y}=X \hat{\alpha}+\hat{b_{i}}+g'(\hat{\mu})(y-\hat{\mu})$, the score test statistics, assuming $\hat{P}\tilde{G}=\hat{P}G$ is:

$$T=G^{t}(Y-\hat{\mu})=G^{t}\hat{P}\hat{Y}=\tilde{G}^{t}\hat{P}\hat{Y=\tilde{G}^{t}(Y-\hat{\mu})}$$

The variance of T is:

$$Var(T)=\tilde{G}W\tilde{G}$$

We estimate this directly for each gene $G$. As shown in [2] this is approximately equivalent to $\tilde{G} \hat{P}\tilde{G}$ but much faster to compute, plus the approximation is conservative.

The effect size $\hat{\beta}$is the natural log of the odds ratio. We can estimate this using the variance component estimate under the null hypothesis.

$$\hat{\beta}=(\tilde{G}W\tilde{G})^{-1}(\tilde{G}\hat{P}\hat{Y})=T/var(T)$$

The standard error of $\hat{\beta}$ is $SE(\hat{\beta})=\left| \hat{\beta}/z \right|$ where $z$ is the z-score corresponding to the p-value divided by 2.

### Simulations

For the β test simulation 3, we sought to generate gene presence/absence matrices, trait values, and age values without using any real sample data. First, we simulated a trait from the binomial distribution with a success probability of 0.5. We assume that there are two strains, one that is correlated with the trait ($odds ratio=2.22$) and one uncorrelated with it. For the correlated strain, we simulated 300 genes at a presence level of 0.5 and an odds ratio of 4.0 for the gene being present given a person has the strain. We simulated one-third of the samples having the uncorrelated strain and modeled this with 250 genes with low binomial presence rates ($p=0.3)$ and an odds ratio of 4.0 for the gene being present given a person has the strain. Then we modeled 300 genes that were “core” across all samples; these were drawn from a binomial with a success probability of 0.8. We additionally simulated 150 genes that were non-strain associated “accessory” genes; these were drawn from a binomial success probability of 0.2. Last, we simulated at least one gene ($G_{y}$) that is even more highly correlated with the trait than is the correlated strain ($odds ratio=2.44$). The more genes in $G_{y}$ (we investigated 1, 2, or 3 genes) and the stronger the relationship between $G_{y}$ and the trait, the higher the parameter 𝜏 will be. The resulting gene presence/absence matrices naturally have a range of different values of $\tau$. Age was randomly generated with parameters similar to the IBD data: $ceiling(rnorm(N,mean=45,sd=15))$. We repeated Simulation 3 with the number of samples varying from 60 to 250.

**References Cited in S1 Text**

1. Chen H, Wang C, Conomos MP, Stilp AM, Li Z, Sofer T, et al. Control for Population Structure and Relatedness for Binary Traits in Genetic Association Studies via Logistic Mixed Models. Am J Hum Genet. 2016;98(4):653-66. Epub 20160324. doi: 10.1016/j.ajhg.2016.02.012. PubMed PMID: 27018471; PubMed Central PMCID: PMCPMC4833218.

2. Zhou W, Nielsen JB, Fritsche LG, Dey R, Gabrielsen ME, Wolford BN, et al. Efficiently controlling for case-control imbalance and sample relatedness in large-scale genetic association studies. Nat Genet. 2018;50(9):1335-41. Epub 20180813. doi: 10.1038/s41588-018-0184-y. PubMed PMID: 30104761; PubMed Central PMCID: PMCPMC6119127.

3. Breslow NE, and Clayton DG. Approximate Inference in Generalized Linear Mixed Models. Journal of the American Statistical Association. 1993;88(421):9-25. doi: 10.1080/01621459.1993.10594284.
