# Supplementary figures and images for: Improved detection of microbiome-disease associations via population structure-aware generalized linear mixed effects models (microSLAM)
Source: PLoS Comput Biol. 2025 May 27;21(5):e1012277. doi: 10.1371/journal.pcbi.1012277 (PMC12136445; doi:10.1371/journal.pcbi.1012277)

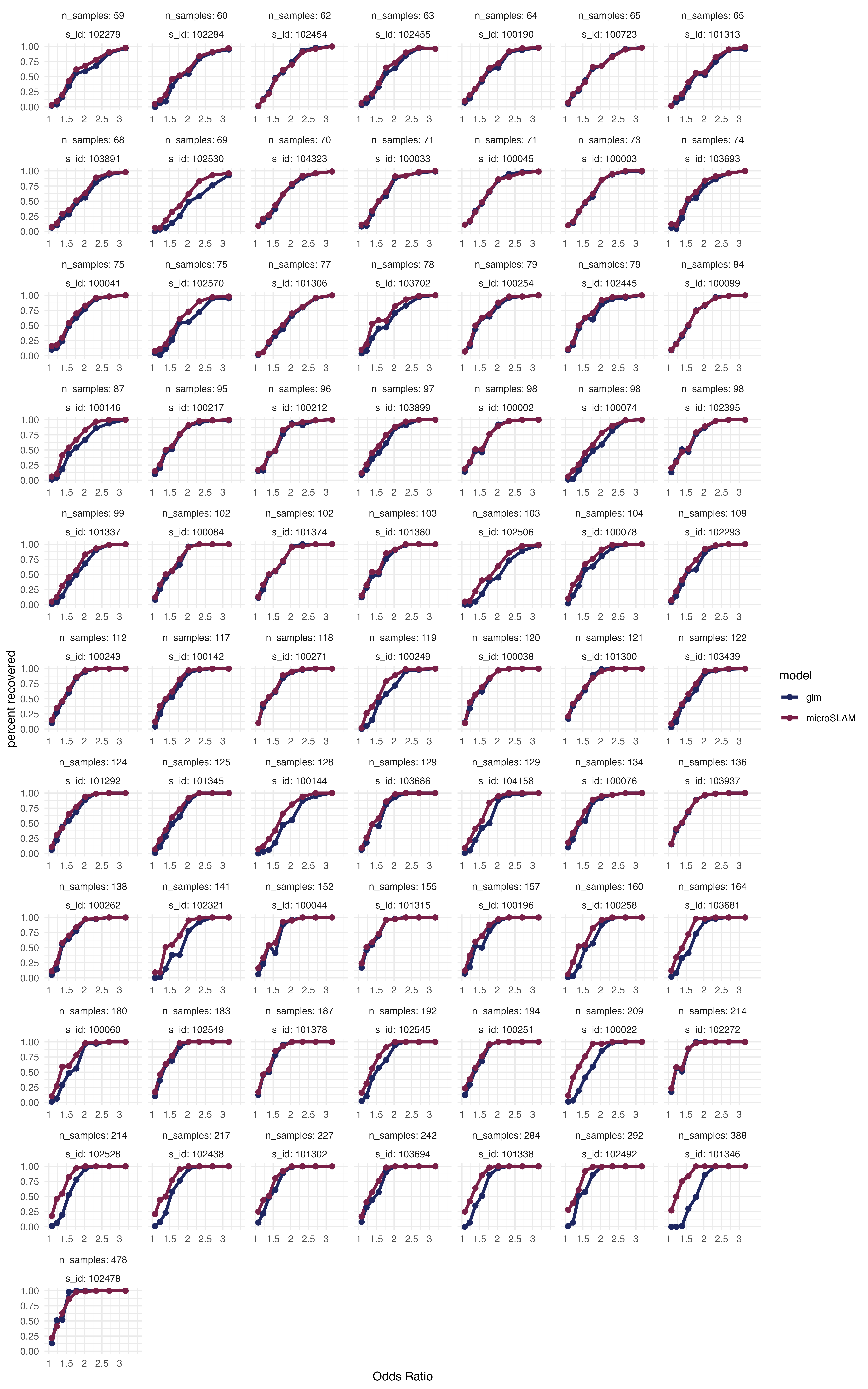

Supplement: S1 Fig — These plots show estimated power of β tests using data from simulation 2 in which a gene presence/absence matrix and binary trait were simulated based on the observed GRMs from the 71 species in the IBD compendium using a range of different effect sizes (odds ratios, horizontal axes). There is one panel per GRM (labeled with species ID), and panels are ordered from lowest to highest sample size. Power was computed as the proportion of positive genes discovered at an empirical localFDR of 0.05 for both microSLAM (red) and glm (blue). As the number of samples increases there tends to be a larger difference between the glm and the microSLAM models. (TIFF) [file pcbi.1012277.s008.tiff]

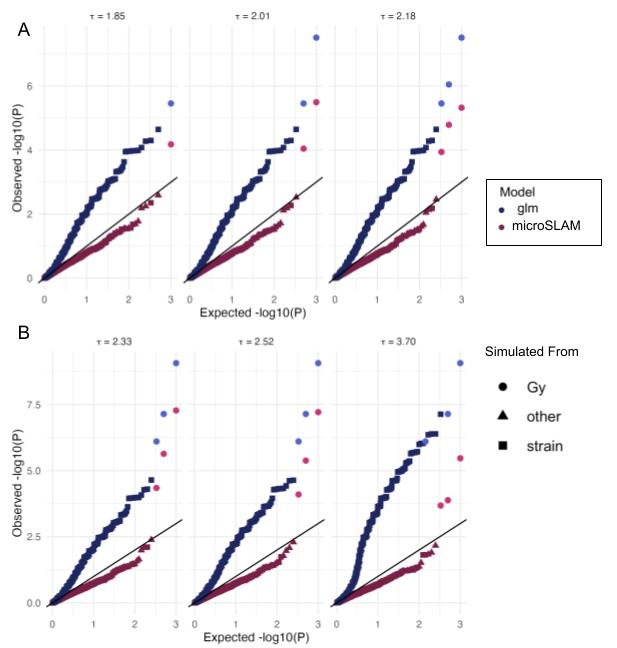

Supplement: S2 Fig — Q-Qplots of β Test results for a simulation with positive genes (Gy; pink circles: microSLAM, light blue circles: glm) plus negative genes that are linked to a strain (strain; squares) or randomly generated (other; triangles) (β test simulation 3, S1 Text). A) Compared to glm (blue), microSLAM (red) better distinguishes the positive genes Gy from those simulated from the strain. The number of positive genes was one (left), two (middle), or three (right). The value of τ increases with each additional gene Gy. B) As the relationship between the strain and y is increased (left to right), the value of τ increases, and the rate of inflation increases for glm. Across different values of τ, microSLAM remains slightly conservative and continues to rank the positive genes Gy highest, indicating high specificity. (TIFF) [file pcbi.1012277.s009.tiff]

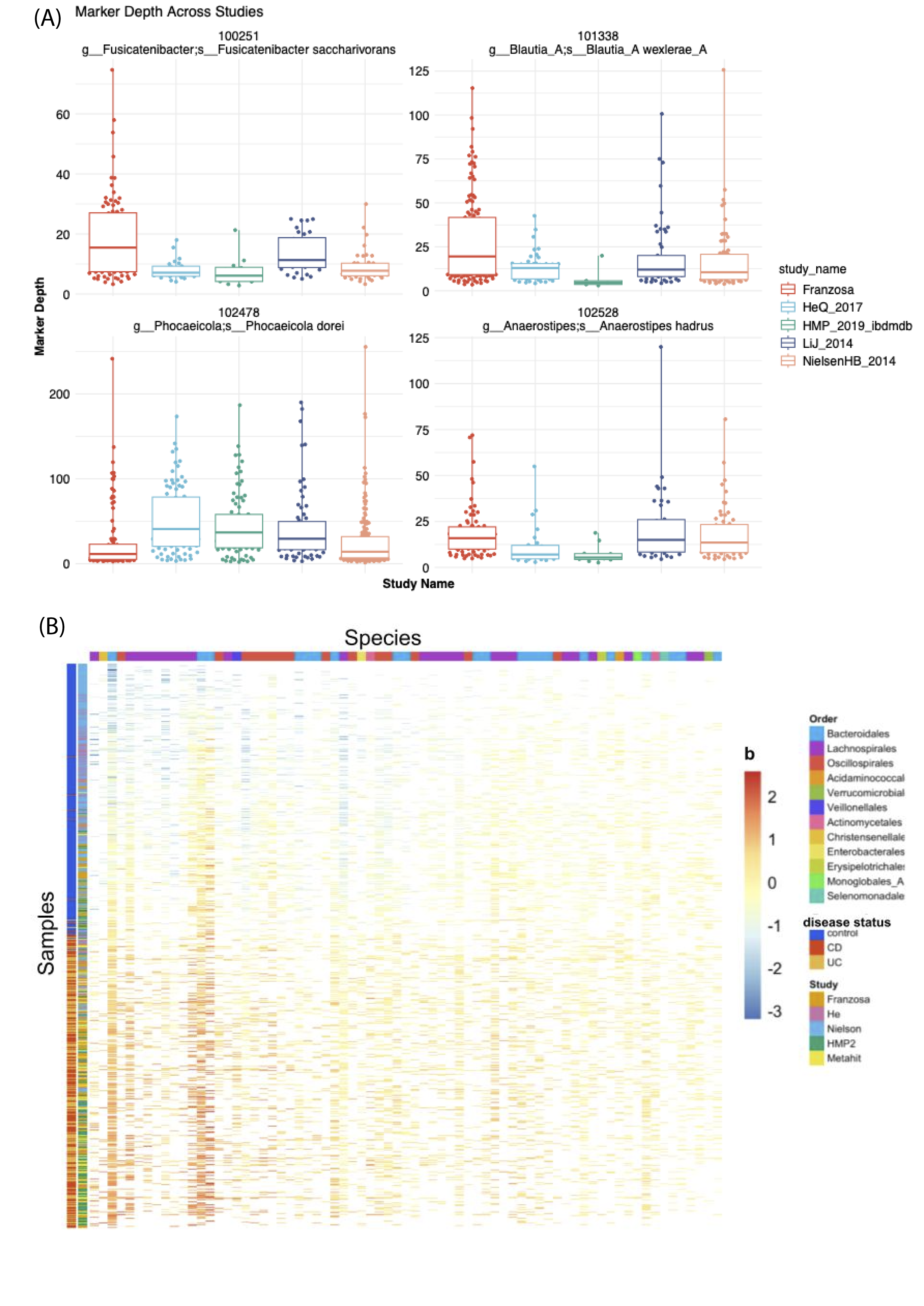

Supplement: S3 Fig — (A) Median coverage of 15 universal, single-copy genes (“marker depth”) in each sample in the IBD compendium, stratified by cohort. We computed marker depth using the MIDAS v3 genes flow. Four representative species are shown. (B) Heatmap of random effect values for each of 71 species across all samples where it was detected in the IBD compendium. The red-to-blue color scale denotes the association between strains and IBD (binary case/control status). Red: strains positively associated with IBD; Blue: strains negatively associated with IBD. The study and IBD subtype of each sample are shown on the left. IBD subtypes: Blue = control, red = Crohn’s disease (CD), yellow = Ulcerative colitis (UC). CD and UC were combined as cases in the microSLAM modeling. Studies: Franzosa (NCBI BioProject PRJNA400072; orange), He (PRJNA398089; pink), Nielsen (PRJEB15371; blue), HMP2 (PRJEB5224; green), MetaHIT (PRJEB1220; yellow). Species are ordered by the standard deviation of b (left = highest standard deviation), where higher standard deviation indicates greater strain diversity that is associated with case/control status. The samples in each column are ordered by lowest to highest average b value. A few studies (e.g., Nielsen) have more controls than others, but there is no systematic relationship between study and population structure. CD and UC tend to have similar distributions of b values (i.e., red and yellow are mixed on the left side bar). While we cannot rule out confounders that were unmeasured in the publicly available data that we could access, these patterns suggest that our findings are not obviously biased by differences in study population (e.g., diet, medical care, geography, type of IBD) that could confound measured associations between case/control status and microbiome strains and genes. (TIFF) [file pcbi.1012277.s010.tiff]

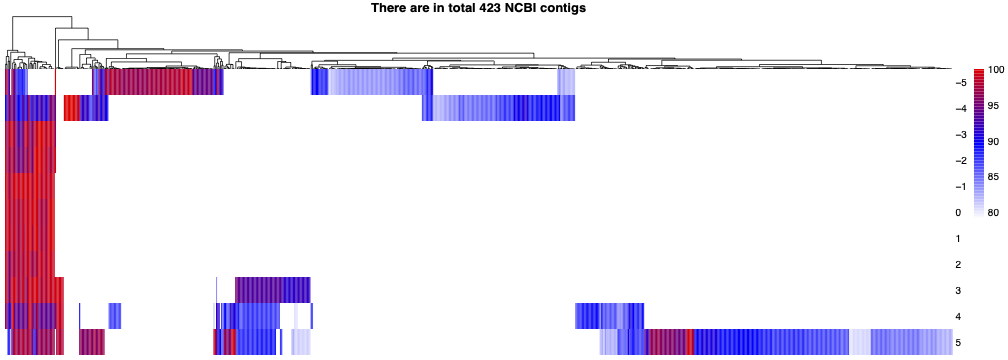

Supplement: S4 Fig — This operon comprises seven genes (occasionally eight genes) that were consistently present or absent together across 53% (49/85) of F. prausnitzii genomes from NCBI. The order and orientation of genes in the operon is conserved. This heatmap shows the genes (rows; position 0 is gfrD, which was significant after localFDR adjustment of microSLAM β test p-values). The other genes were significant before localFDR adjustment and are indexed relative to gfrD in the heatmap. Columns represent 423 contigs from 85 F. prausnitzii high-quality NCBI genomes. The color of the heatmap shows the blastn sequence similarity of the gene sequence in the contig compared to the sequence in the F. prausnitzii reference genome used in our microSLAM analysis (red=highest similarity, white=no significant match). The seven genes in the operon (middle rows of the heatmap) have high sequence similarity when they are present and are present together (red on left), whereas flanking genes are more variably present and have lower sequence identity (blue in top and bottom rows). (TIFF) [file pcbi.1012277.s011.tiff]

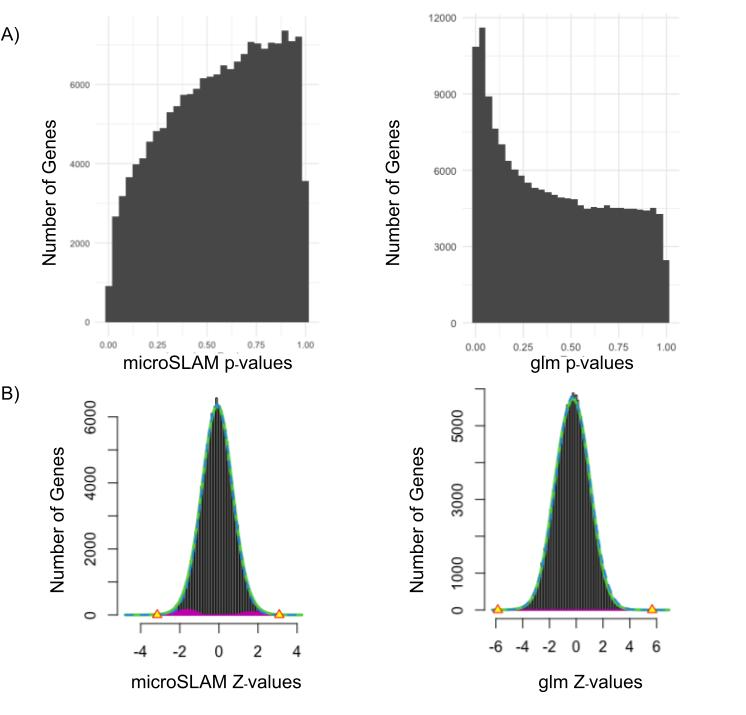

Supplement: S5 Fig — (A) Histogram of p-values for microSLAM’s β test (left) and glm (right). (B) Output from localFDR showing the distribution of the null z-values (green) versus the distribution of the z-values that do not follow the null (pink). Yellow triangles denote the z-value thresholds corresponding to a localFDR of 0.2. (TIFF) [file pcbi.1012277.s012.tiff]

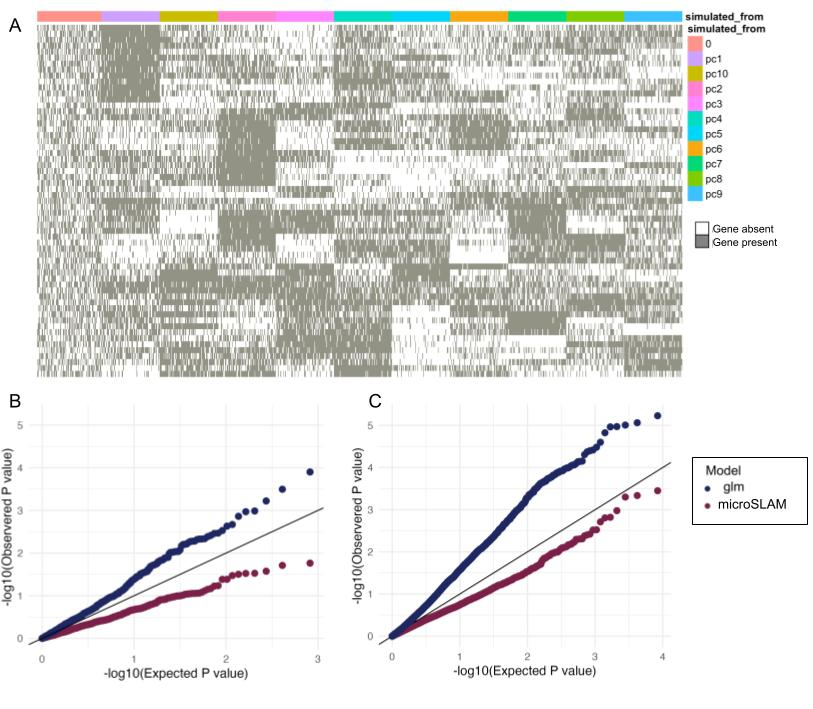

Supplement: S6 Fig — (A) Simulated gene presence/absence matrix based on the GRM of Bacteroides thetaiotaomicron plotted as a heatmap (grey = gene present in a given sample, white = gene absent). Genes are in columns and are labeled according to how they were simulated (0 = random, pc1–10 = using one of the first 10 principal components of the observed GRM for B. thetaiotaomicron. This presence/absence matrix has some population structure (estimated τ = 2.30), but no genes were simulated to be associated directly associated with the trait which is defined by the first two PCs. (B) Q-Qplot of p-values from all genes not from PC1 or 2 from microSLAM’s β test (red) and glm (blue) applied to the simulated gene presence/absence matrix in (A). There is a much higher error rate for the glm model. On the other hand, microSLAM is overly conservative (i.e., underpowered). (C) Q-Q plot for microSLAM’s β test (red) and glm (blue) applied to the observed B. thetaiotaomicron gene presence/absence matrix from the IBD compendium. The trends are very similar to those in the simulation. (TIFF) [file pcbi.1012277.s013.tiff]

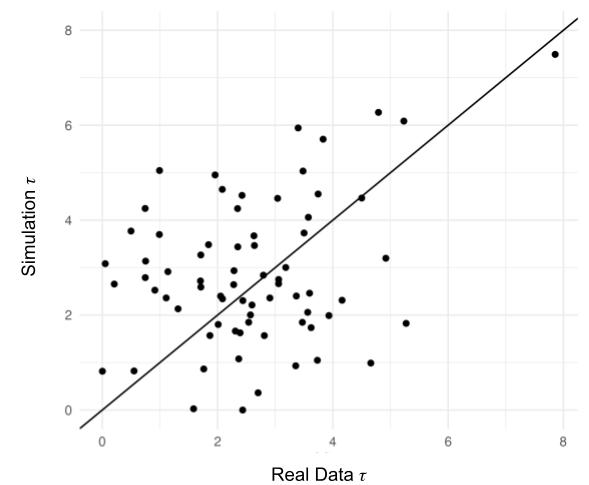

Supplement: S7 Fig — In the β Test Simulation 1 and 2 set up, we generated gene presence/absence matrices using the observed GRMs for the 71 species in the IBD compendium. Our objective was to generate simulated data that was similar to but not identical to the observed data (Methods). This scatter plot shows the τ values estimated by microSLAM on the simulated data (y axis) compared to the corresponding τ values estimated from the real data in the IBD compendium (x axis). The n τ from the simulation cover a similar range of values as those from the real data while not being highly correlated. (TIFF) [file pcbi.1012277.s014.tiff]
