# Supplementary material for: Improved detection of microbiome-disease associations via population structure-aware generalized linear mixed effects models (microSLAM)
Source: PLoS Comput Biol. 2025 May 27;21(5):e1012277. doi: 10.1371/journal.pcbi.1012277 (PMC12136445; doi:10.1371/journal.pcbi.1012277)
Supplement: Attachment — Submitted filename: MicroSLAM-Response.docx [file pcbi.1012277.s016.docx]

We thank the reviewers for their thoughtful and constructive feedback, which has helped us to improve our manuscript. Major changes in this revision include:

1. Revising the Results, Methods, and GitHub code repository to provide a clearer description of microSLAM, including what is novel about it and how to use it.
2. Adding a covariate to the microSLAM models to account for potential unmeasured batch effects that could confound our IBD association analyses.
3. Clarified that microSLAM is not a causal inference method.
4. Added text discussing cross-species function-based approaches, quality control, and metagenome-assembled genomes.
5. Fixed typos and confusing phrases to improve readability.

Below is a point-by-point response to reviewer comments (in black font) with our replies in red. Changes are highlighted in the manuscript and supplement using blue font.

**Reviewer #1:**

First of all, I like the main idea of the manuscript, which applies statistical methods from GWAS studies to metagenomics—a novel approach that I believe will be useful for researchers in microbiome studies.

We thank the reviewer for their positive assessment regarding our modeling approach and the utility of microSLAM.

That said, the manuscript currently falls short in clarity, particularly concerning the statistical model, which is the primary focus of the work. The model is challenging to understand due to confusing symbols and unexplained technical terms across the manuscript. The methodology part of the manuscript is hard to read. Below are some examples:

This is very helpful feedback. To address it, we completely re-wrote sections of Results and Methods (“MicroSLAM framework”), added a simple description of the approach as a new first subsection of Results (“Overview of microSLAM”), added a Methods subsection with a step-by-step description of a microSLAM analysis (“microSLAM Runtime Checklist”), and provided definitions for technical terms. Specific changes are described for the provided examples.

We appreciate the reviewer’s input on how to improve the clarity of our work. Please let us know if additional clarifications are needed.

For example, the author first mentioned tau that refers to population structure in line 571, 584 (tau test actually mentioned at the beginning of the result, line 127). However, it is only in line 594 that the author clarifies that tau represents the variance of the random effects.

This is a great suggestion. In this revision, we define tau clearly and much earlier. We also now avoid using tau to refer to population structure, instead restricting its use to describing the model parameter that quantifies the variance of the random effects.

Line 611 is another example: “Similar to the strategy used in SAIGE [27], we directly calculate the score statistic for each gene by fitting the covariate and population structure adjusted genotype vector to the phenotype. Doing a direct computation given the random effect is an efficient strategy to reduce compute time;” Terms like “score statistic” and “direct computation” are presented here but without explanation. Likewise, it’s unclear what is meant by “fitting the covariate- and population-structure-adjusted genotype vector to the phenotype.” These terms seem directly drawn from the SAIGE paper, but readers won’t understand them without reading the SAIGE paper.

We agree that these phrases were confusing, and they did not add a lot of useful information beyond what one can read in the SAIGE manuscript if they are interested in the underlying estimation procedure. Hence, we deleted much of this paragraph. The new text reads:

“To test the null hypothesis that a given gene’s $\beta=0$, we follow the strategy employed in SAIGE ^29^, which uses a score statistic and does not re-estimate $\alpha$ or *b*. By only fitting Model 1 once per species, this approach is computationally efficient.”

Compared to the confusing main text, the supplementary text is much better but still not clear. In general, I had to rely heavily on the references cited within the manuscript to fully understand the methodology.

We thank the reviewer for making this extra effort, and we’re sorry that our methodology was not clearer in the initial submission. To address this, we moved some text from the supplement into Results and augmented this with new stand-alone text that explains microSLAM in simple terms (see new subsection “microSLAM Framework” at the start of Results). In a new subsection (“Overview of microSLAM”), we provide more detail, using equations to be explicit about each modeling step. All terms in the equations are clearly defined. Finally, in Methods, we added a step-by-step description of the process of running microSLAM (“microSLAM Runtime Checklist”). We hope these and other changes throughout the manuscript have helped to reduce confusion.

The main statistical model is clearly closed to SAIGE [27] and GMMAT [26], which is okay even if the same method is used. However, the authors do not clearly indicate what they have adapted from previous work and what is new in their own implementation of GLMM. If authors did modify the original method, a comparison between the new framework and SAIGE or GMMAT would be needed.

We welcome the chance to clarify how our work builds on SAIGE and GMMAT. We did modify the SAIGE model in a few ways, although our overall strategy is adapted from SAIGE. The revised text explicitly states that microSLAM adapts SAIGE’s core framework with modifications that enable GLMMs to be applied to microbiome data. These include:

- Replacing the SNP-based genetic relatedness matrix (GRM) with a gene presence/absence based GRM. This involved writing code that uses a binary genotype (0/1) rather than the usual count of the number of minor alleles (0/1/2). We also implemented (1 - Manhattan distance) as the similarity measure for computing the GRM.
- Adding code to loop over species and, for each species, to adjust the analysis to use only those samples where that species is detected.
- Using a non-parametric permutation approach in the $\tau$ test, rather than a likelihood ratio test. This is important because assumptions of the likelihood ratio test may not be met in microbiome datasets.

We also clearly defined the $\tau$-test and $\beta$-test as separate steps in microSLAM, so that readers can understand each step of microSLAM's methodology independently, even if they are not familiar with SAIGE.

For further improvement, I would recommend collaborating with a statistician or biostatistician to rewrite the methodology section. Alternatively, the authors might consider revising the manuscript as an analysis paper that highlights the novel analysis pipeline.

We took the reviewer’s advice to rewrite the methodology sections.

**Reviewer #2:**

It was a pleasure to read the manuscript from Goldman and Co. The language is clear and well written. I have to admit, that I have no experience yet with running pangenome analysis on populations of different species found in metagenomes with tools such as MIDAS2 or 3. So this was a bit of a learning curve for me, to read this manuscript. I believe the whole approach of identifying microbiome species and their genes that have a significance in the occurrence of disease is really interesting. The microSLAM package seems to do that pretty well and I was pleasantly surprised by the finding of genes (the fructoselysine operon) that seem to be more mobile than one expects from a group of genes involved in carbon metabolism.
The structure of your manuscript is good in my eyes and I like the use of publically available datasets instead of using artificial datasets to show how the method works. That makes this manuscript on an R-package a lot more interesting for a microbiologist as myself.
For this manuscript, I find that I have very little to comment on and below you only find a comment on the installation instructions of microSLAM and several minor comments.

We are grateful for this positive feedback! We sincerely appreciate the reviewer’s thoughtful comments on our manuscript. We are glad to hear that the methodology and application of microSLAM were of interest. We also appreciate the reviewer's acknowledgment of the clarity of our manuscript and the value of using publicly available datasets. Below, we address the specific comments and suggestions.

Installation comment.
One of the first things I did was to try to install microSLAM in R-studio. It turned out, that I needed an extra library in order to be able to install microSLAM with the command install_github which was not on your github instructions. That library is "remotes". check if that is needed. After that, the installation went fine, and I could run the example that you made in the MicroSLAM repository.

We thank the reviewer for the feedback on the installation process! While the remotes package can be used to install packages from GitHub, our recommended approach is to use devtools, which provides broader functionality for R package development, including installation, testing, and debugging. To clarify this in the installation instructions, we have updated the documentation on GitHub to explicitly specify:

```

install.packages("devtools")

library(devtools)

devtools::install_github('pollardlab/microSLAM')

library(microSLAM)

```

Minor comments.
Lines: 276 - 278: This sentence seems rather vague and is not complete in my opinion. There are DNA extraction methods which can cause batch effects, as well as sequencing library preparation methods. Do you have a reference for that statement?

We appreciate this suggestion and have revised the sentence for clarity. The updated sentence now reads:

“Beyond biological variability, variations in DNA extraction methods, and sequencing library preparation protocols can introduce batch effects in microbiome studies, potentially affecting microbial profiling results.”

We also added two references at the end of this sentence to support the claim that DNA extraction and library preparation can introduce batch effects in microbiome studies:

- <https://journals.asm.org/doi/10.1128/spectrum.00090-22>;
- <https://genomebiology.biomedcentral.com/articles/10.1186/s13059-024-03401-9>

Line: 329-330 This reads weird: " ... addition to the understand these genes ..."

We have completely rewritten the sentence for better readability:

“To be detected after adjusting for population structure, trait-associated genes must be evolving somewhat independently of the genes that distinguish strain lineages, with mobile genetic elements being particularly strong signals in the cohorts we analyzed.”

Line 469 - 470: Here is something missing or not right in the text. The sentences do not make sense to me.

Thanks for catching this. We re-wrote this sentence as follows:

“We did investigate if any microSLAM discoveries were mostly driven by one subtype or the other, and we observed very few examples of trait-strain or trait-gene associations that were attributable to only Crohn’s samples or only ulcerative colitis samples. One significant gene cluster from *Phascolarctobacterium faecium* (GUT_GENOME040547_00268) was found to be positively associated with IBD (β test) but was only present in Crohn’s patients.”

Line 484 - 487: I wonder, could you also use MAGs derived from the same samples to test these findings? For instance identifying the gene operon from the MAGs? Or is could that be fraught with other issues?

Thanks for the suggestion. Cross-mapping is indeed a challenge in read-mapping-based analyses, as it can lead to genes from the same operon showing varying statistical significance. For example, in our study, only one gene from the PTS operon had a significant p-value after multiple testing correction, even though all genes in the operon had nearly significant adjusted p-values and we found strong evidence in genome assemblies that the whole operon tends to be either present or absent. We attribute this variability in p-values to a mix of random read sampling and cross-mapping. This underscores the need for a second source to validate microSLAM results. We therefore used isolate genome assemblies and metagenome-assembled genomes (MAGs) to validate the PTS operon.

To address the reviewer’s comment, we added text to emphasize that validation using genome assemblies (isolates and MAGs) is a general approach that can be applied to any microSLAM gene hits. High-quality genome assemblies are essential in this context, as fragmented metagenome-assembled genomes (MAGs) can misrepresent operon structures or fail to capture their presence consistently across strains. For metagenomes from well-studied environments, MAGs available in databases like NCBI provide a valuable resource. However, for species or strains that are underrepresented in databases such as NCBI or GTDB, assembling MAGs directly from the samples is crucial. We have updated the manuscript to reflect this recommendation and to emphasize the complementary role of MAGs in validating microSLAM analyses.

**Reviewer #3**

The current study describes microSLAM a novel method for carrying out microbiome-phenotype association studies. While the work first pinpoints individual trait-associated strains, its novelty is the focus on the specific microbial genes associated with the trait. This is an important improvement over the state of the art, as microbiome HGT requires a fairly loose definition of a strain and makes identification of culprits difficult (and often non-reproducible) across studies. The paper is well written and timely. However, some problems of the manuscript are detailed below:

We thank the reviewer for their positive feedback regarding the novelty, timeliness, and utility of microSLAM! We also appreciate the acknowledgement that the manuscript is well written. We welcome the opportunity to address the reviewer’s concerns below.

Major
1. The stated goal of the study seems to be the finding of trait causality rather than biomarkers. If indeed so, it is unclear why the associated organism needs to be pinpointed first (tau test). This point of view suggests that “genes” and not “functions” are the basic units of causality. However, individual genes, specific to bacterial lineages, may or may not reflect all of the specific function F within the microbiome, which these genes carry out. That is, species A and unrelated species B may perform the same function F using different genes. In the absence of the function to phenotype mapping, identified gene-phenotype associations are incomplete and biased by our ability to reconstruct strains. I would suggest that some annotation of metagenomic genes (or directly reads!) with associated functions (e.g. EC numbers using HUMANN or MGNIFY pipeline, or a wider set of fusion functions using fusionDB or mi-faser) would be more relevant for the type of analysis that microSLAM wants to bring forth. Incidentally, not requiring species baselines would allow for a much wider analysis of genes/functions in the microbiome. I realize that the above analysis may not be possible given the ready-made tool. However, I insist on the inclusion of some discussion of the merits in establishing causality of the described approach vs. a function-focused, population structure one. If finding biomarkers (not causality) is the goal – this should be made more clear in the text.

We appreciate the reviewer’s insightful feedback on the distinction between causality and biomarker identification, as well as the methodological framework underlying our approach. Below, we address these concerns and clarify the rationale behind our methodological choices.

First, microSLAM is a lineage-specific gene-trait association test that corrects for strain-level population structure. This approach enables the identification of genes whose presence or absence is significantly associated with a host trait, making them promising candidates for discovering causal mechanisms. However, microSLAM does not directly establish causality. We acknowledge that introducing the concept of causality early in the manuscript may have created confusion. Therefore, we revised the manuscript to clarify that microSLAM gene hits would require further follow-up studies to establish causality:

“Genes like this are still only biomarkers for the trait (i.e., statistical associations), but they are promising candidates for follow-up studies aimed at discovering causal mechanisms through which microbes modify host health and treatment responses.”

After this sentence, we defer all further comments regarding causality until the Discussion section, where we emphasize the need for experimental validation beyond statistical associations.

Second, we appreciate the reviewer bringing up function-based approaches, like HUMAnN and Mgnify. These tools categorize genes into metabolic pathways and enzyme families, effectively capturing functional redundancy across species. While these methods are useful for understanding broad metabolic capabilities, they rely on existing annotations, which may fail to capture poorly characterized, recently acquired, or mobile genetic elements. Such genes can play important roles in strain-level adaptations (e.g. antibiotic resistance or host immune evasion). Although cross-species function-to-phenotype mapping is valuable, and often better powered than within-species approaches, it cannot replace species-level gene-trait associations. MicroSLAM is an important complementary approach, particularly in cases where lineage-specific genetic variation influences host interactions in ways that functional databases may not fully capture or when follow-up experiments require identifying specific strains carrying genes of interest. To introduce the concept of function-to-phenotype mapping and highlight how it differs from microSLAM’s lineage-specific gene-trait associations, we have revised the Introduction by adding a new paragraph. This addition provides context on how function-based approaches contribute to microbiome studies while leading into the rationale for species-level gene-trait associations as a complementary and necessary method.

2. It is unclear how the identified associations can be verified. While simulations are meaningful, they do not capture the specific biology of disease. In the absence of experimental data (and limited gene info as in Fig 4E), an option for validation is to separate the development set of metagenomes into individual cohorts (and do cross-validation) or to collect additional data to explore whether the strains and/or genes identified for one set can be extracted for the others. How reproducible are the results is an important question, unless the claim is that IBD in different cohorts of people is caused by different genes. If this is indeed the claim, it should be made significantly more clear.

We appreciate the reviewer’s concerns regarding the verification and reproducibility of our identified associations, particularly in the absence of experimental validation. Simulations play a crucial role in evaluating the statistical performance of microSLAM, ensuring proper false positive control and accounting for population structure effects. However, we acknowledge that simulations alone cannot fully capture the biological complexity of disease. Below, we detail our approach to addressing these challenges.

We initially were excited about performing a leave-one-dataset-out (LODO) analysis, as suggested by the reviewer. As we dug into implementing this, we realized that limited sample sizes within cohorts, combined with class imbalance in some cohorts, would make interpreting the results of LODO difficult. Even if cohorts were drawn from populations with similar $\tau$ or $\beta$ values, these differences in sampling alone could make it impossible to detect associations. Hence, if we observed lack of replication in LODO, we would not know whether to attribute it to these sampling differences or to true lack of consistent associations. Specifically, our simulations show that microSLAM requires a minimum sample size of 60 (with equal numbers of cases and controls), with preferably 100 or more for robust detection (especially if the case:control ratio differs from 50:50). Given these constraints in our current dataset, conducting thorough LODO evaluations proved impossible. Although 14 species met the criteria for training and testing in a LODO framework, we observed a highly unbalanced case vs. control ratio in many species-cohort combinations (recall that the samples used for a given species are only those where the species is robustly detected). This imbalance makes it difficult to ensure statistical power and comparability across cohorts. Hence, we moved on to other approaches to address the reviewer’s comment.

To assess whether cohort-specific effects influenced our results, we next examined species marker gene coverage across cohorts, estimated via MIDAS3 (which uses 15 universal, single-copy marker genes). This statistic is correlated with species abundance, for example as estimated using a standard Kraken2+Bracken pipeline. But it also captures some technical sources of variation, such as sequencing depth and unmeasured confounders that may contribute to batch effects. Our analyses revealed significant variations in marker gene coverage across studies, suggesting that cohort effects could indeed confound gene-trait associations. This figure has been added to the supplementary materials (S3 FigA). It shows distributions of marker depth for four example species across the cohorts:

To mitigate this, we updated the models throughout the manuscript by adding maker gene coverage as a covariate. This approach indirectly accounts for cohort-specific biases without explicitly controlling for the study name, thereby retaining statistical power (one additional parameter versus four additional parameters), while still addressing inter-study variation.

We also added new text acknowledging that, beyond species abundance and sequencing depth, additional cohort-specific factors (e.g., host genetics, diet, medication usage, etc) could also contribute to confounding. The microSLAM package enables additional covariates to be added when they are measured. However, these variables were not available in the publicly available IBD datasets, limiting our ability to directly account for them. Given this, adding marker gene coverage as a covariate represents a practical compromise. To evaluate the effect of this addition, we compared the τ (tau) values from models with:

- Age as the only covariate
- Age and marker gene coverage (marker_depth) as covariates

We found that:

- Out of 71 species, only 3 species showed a significant change in tau values
- Out of 83 significant genes, 49 (including the PTS operon hit) remained significantly associated with the trait in the updated model. The beta coefficients were highly correlated between the two model (R=0.998).

This figure shows the beta coefficient estimates from the two models:

These results suggest that while cohort effects exist, the core findings remain largely robust to this adjustment. We thank the reviewer for pointing out the importance of adjusting for cohort variability. This suggestion led to an improvement in our modeling approach (adding species’ marker_depth to the models), which helps to mitigate cohort variability and improves the robustness of our findings.

Minor
1. I am curious how microSLAM performs across vs within different cohorts, where batch effects may be an issue. In the analysis, 710 metagenomes form 5 studies are used. Would using one be better? Was there any error correction? Some discussion on the topic of sequencing depth, techniques differences, etc. in establishing microbiome structure would be helpful. On that same note, is there ever a time when two different cohorts studying the same disease can be unified for more power?

This is an important point. In the near future, as sample sizes grow, it will be possible to apply microSLAM to individual studies (with limited batch effects) and compare results across studies. At the present time, however, we found that it is necessary to combine data across studies to achieve sufficient power. In addition, our primary goal is to uncover microbial genes that are consistently associated with disease across multiple studies rather than to identify study-specific associations. The IBD analysis in this study likely has false negatives due to batch effects, but we do not expect batch effects to cause false positives, making our hits robust candidates with signals that exceed batch effects.

Combining multiple studies is possible when the studies have compatible metadata and similar disease definitions. In this study, we combined datasets based on IBD case/control status, allowing microSLAM to achieve the statistical power required for MWAS, as single studies lack sufficient sample sizes for robust gene-trait association testing. While merging datasets introduces heterogeneity, we accounted for this by adding species abundance as a covariate, indirectly controlling for cohort-specific effects while preserving statistical detection power, as outlined in the previous response.

Additionally, we implemented stringent QC to ensure reliable MWAS results, including (1) select species-sample pairs with genome-wide vertical coverage higher than 5X, and consistent with marker gene coverage, and (2) filter out extremely common or rare genes.

However, we acknowledge that other unmeasured cohort-specific factors such as differences in DNA extraction protocols, sequencing platforms, or host-associated variables (e.g., diet, medication) may still introduce variability. This important caveat has been elaborated upon in the Discussion section.

2. There are five issues/observations/concerns with using microSLAM that were raised by the authors in the paper discussion. These make using the tool difficult in a generic situation, i.e. I have a bunch of metagenomes and I want to know which genes are associated with a metagenome-specific trait. Please provide a specific description (almost like a help document) for what is expected as the data input so that the results can be trusted.

This is a fantastic suggestion! First, we shortened the discussion of limitations somewhat so that it is a bit less intimidating, and we clarified that several of the limitations are not specific to microSLAM but rather issues in any metagenomic analysis. Second, we really appreciate the reviewer’s request for a detailed guide on how to apply microSLAM in a generic metagenomic analysis setting. In the Results, we added a step-by-step walk through of how to perform a microSLAM analysis from QC through to statistical testing and visualization. For each step of microSLAM, the goal is explicitly stated. This new Methods text (“microSLAM Runtime Checklist”) includes a structured description of the data input requirements, preprocessing steps, and quality control criteria necessary for ensuring reliable results when using microSLAM. In addition, we refer users to the code examples provided in the microSLAM GitHub repository (<https://github.com/pollardlab/microSLAM>).

3. I would argue that the word several here “several human diseases [2–5]” is not necessary. It implies that only a few diseases are microbiome associated, but I would argue that many are.

We appreciate the reviewer’s suggestion. To address it, we change “several” to “numerous”.

**Editorial Comments**

In addition, we made several minor modifications to address feedback regarding journal formatting and requirements.

1) We note that you have uploaded Figure 1 to Figure  5 as other files. Please upload all main figures as separate Figure files in .tif or .eps format. For more information about how to convert and format your figure files please see our guidelines: 
<https://journals.plos.org/ploscompbiol/s/figures>

We uploaded Figures 1-5 as .eps files.

2) Please upload a copy of Figure 1 to 5 which you refer to in your text  on the manuscript file. Or, if the figure is no longer to be included as part of the submission please remove all reference to it within the text.

We uploaded Figures 1-5 as .eps files and confirmed that we refer to all of them in the text.

3) Thank you for stating "No competing interests"

Please include a completed 'Competing Interests' statement in the text box when submitting your production task, including any COIs declared by your co-authors, written in full sentences. 
If you have no competing interests to declare, please state "The authors have declared that no competing interests exist". You may also provide an updated statement via email.

Done

4) Please ensure that the affiliations of the authors [ Chunyu Zhao, Katherine S Pollard] listed on the manuscript title page do exactly match with the affiliations provided in the online submission form
NOTE: Affiliations should include a department (if applicable), an institution, a CITY, and a country

Done

5) We have noticed that you have uploaded Supporting Information files, but you have not included a list of legends. Please add a full list of legends for your Supporting Information files after the references list.

As suggested, we added a full list of legends for Supplemental Tables and Supplemental Figures in the Supporting Information files. This appears after the references list.

6) Some material included in your submission may be copyrighted. According to PLOSu2019s copyright policy, authors who use figures or other material (e.g., graphics, clipart, maps) from another author or copyright holder must demonstrate or obtain permission to publish this material under the Creative Commons Attribution 4.0 International (CC BY 4.0) License used by PLOS journals. Please closely review the details of PLOSu2019s copyright requirements here: PLOS Licenses and Copyright. If you need to request permissions from a copyright holder, you may use PLOS's Copyright Content Permission form. 
Please respond directly to this email and provide any known details concerning your material's license terms and permissions required for reuse, even if you have not yet obtained copyright permissions or are unsure of your material's copyright compatibility. Once you have responded and addressed all other outstanding technical requirements, you may resubmit your manuscript within Editorial Manager.  
Potential Copyright Issues: 
i) Figure 5c. Please confirm whether you drew the images / clip-art within the figure panels by hand. If you did not draw the images, please provide (a) a link to the source of the images or icons and their license / terms of use; or (b) written permission from the copyright holder to publish the images or icons under our CC BY 4.0 license. Alternatively, you may replace the images with open source alternatives. See these open source resources you may use to replace images / clip-art:
- <https://commons.wikimedia.org>
- <https://openclipart.org/>.
ii) We note that [Figure 1] is created through BioRender. Please confirm that you hold a Premium account and provide a pdf copy of the CC BY 4.0 Licence as provided by BioRender. For instructions on how to generate a CC BY 4.0 license for your figure, please see the guidelines here: <https://help.biorender.com/hc/en-gb/articles/21282341238045-Publishing-in-open-access-resources>. 
If you are using the free assets from BioRender, we are unable to publish these images as they are licenced under a stricter licence than CC BY 4.0. In this case we ask you to remove the BioRender images and replace them with open source alternatives.

We used BioRender to create Figure 1a and Figure 5c. We have a premium account. PDF copies of the licenses from BioRender has been uploaded.

7) Thank you for stating "Reference genomes and metagenomic data analyzed for this study are available in public repositories as described in the Methods."
Please amend the statement and include full link to the data
NOTE: Please note that your Data Availability Statement is currently missing the repository name, and the DOI/accession number of each dataset OR a direct link to access each dataset. If your manuscript is accepted for publication, you will be asked to provide these details on a very short timeline. We therefore suggest that you provide this information now, though we will not hold up the peer review process if you are unable.

We edited the Data Availability statement to read as follows:

“Reference genomes were obtained from the UHGG database version 2, available from the Mgnify FTP site (http://ftp.ebi.ac.uk/pub/databases/metagenomics/mgnify_genomes/) and also available from the European Nucleotide Archive under study accession ERP116715. Additional Faecalibacterium prausnitzii genomes were downloaded from NCBI. Supplemental Table 5 contains a full list of the Faecalibacterium prausnitzii NCBI accession numbers.

Metagenomic sequencing data were obtained from NCBI. Supplemental Table 1 contains a full list of study accession numbers. Supplemental Table 6 contains a full list of sample accession numbers.”

8) Please amend your detailed Financial Disclosure statement. This is published with the article. It must therefore be completed in full sentences and contain the exact wording you wish to be published. 
1) State the initials, alongside each funding source, of each author to receive each grant. For example: "This work was supported by the National Institutes of Health (####### to AM; ###### to CJ) and the National Science Foundation (###### to AM)."
2) State what role the funders took in the study. If the funders had no role in your study, please state: "The funders had no role in study design, data collection and analysis, decision to publish, or preparation of the manuscript."
3) If any authors received a salary from any of your funders, please state which authors and which funders.. 
If you did not receive any funding for this study, please simply state: u201cThe authors received no specific funding for this work.u201d 

Done.

9)  Please ensure that the funders and grant numbers match between the Financial Disclosure field and the Funding Information tab in your submission form. Note that the funders must be provided in the same order in both places as well.
Currently,  " The Chan Zuckerberg Biohub San Francisco, and Gladstone Institutes" are missing from the Funding Information

Done.
